# Deciphering the circadian rhythm in colorectal cancer: a bibliometric analysis of research landscape and trends

**DOI:** 10.3389/fonc.2025.1591257

**Published:** 2025-06-16

**Authors:** Linzi Chen, Zhongjie Wang, Ningkun Xiao, Jinhui Liu, Yuhan Tao, Sifang Zhang

**Affiliations:** ^1^ Department of Integrated Traditional Chinese & Western Medicine, The Second Xiangya Hospital, Central South University, Changsha, Hunan, China; ^2^ Department of Immunochemistry, Institution of Chemical Engineering, Ural Federal University, Yekaterinburg, Russia; ^3^ Laboratory for Brain and Neurocognitive Development, Department of Psychology, Institution of Humanities, Ural Federal University, Yekaterinburg, Russia; ^4^ The Second Xiangya Hospital, Central South University, Changsha, Hunan, China

**Keywords:** colorectal cancer, circadian rhythm disruption, tumor microenvironment, colorectal cancer intervention, bibliometric

## Abstract

**Introduction:**

Colorectal cancer (CRC) is a leading cause of global cancer mortality, increasingly linked to circadian rhythm disruption—a critical yet underexplored driver of tumorigenesis.

**Methods:**

This bibliometric analysis evaluates 374 publications from the Web of Science Core Collection (1999–2024) using VOSviewer, CiteSpace, and Bibliometrix to map global research trends.

**Results:**

Annual publications surged post-2016, peaking in 2021, reflecting intensified focus on circadian-CRC interactions. The United States led in output (122 publications, H-index 46), followed by France (76 publications) and China (49 publications), with the Netherlands achieving the highest citation impact (88.06 citations per publication). French institutions, notably Assistance Publique–Hôpitaux de Paris (APHP), dominated translational research, while foundational studies by Levi et al. on chronomodulated chemotherapy remained pivotal. Keyword analysis identified “circadian rhythm” and “colorectal cancer” as core themes, with “inflammation” and “inflammatory bowel disease” showing significant citation bursts post-2014. Co-citation networks bridged molecular chronobiology (Science, PNAS) and clinical oncology (Cancer Research), though mechanistic studies prioritized clock genes (e.g., BMAL1, PER2) over environmental disruptors. Clinically, aligning chemotherapy with circadian rhythms reduced severe toxicity by 40% in metastatic CRC, yet gaps persist in biomarker validation and monitoring tools. Epidemiologically, shift workers faced a 20–30% elevated CRC risk, correlating with PER2 silencing in 45% of tumors and NF-κB/STAT3 pathway activation.

**Discussion:**

Future research should integrate AI-driven circadian profiling, global collaboration, and trials targeting circadian-immune-metabolic axes to advance precision chronotherapy. This study underscores circadian biology as a cornerstone of CRC management, advocating strategies that harmonize molecular insights with ecological relevance to improve outcomes.

## 1 Introduction

Circadian rhythms represent an evolutionarily optimized timekeeping machinery that orchestrates physiological and molecular processes within 24-hour cycles, synchronizing organisms with environmental zeitgebers like light-dark cycles and feeding patterns (1). Beyond regulating sleep-wake cycles, these biological oscillators regulate critical cellular functions including DNA repair, metabolism, and immune surveillance—processes now recognized as intricately linked to cancer pathogenesis (2, 3). The therapeutic rationale for chronomodulated chemotherapy stems from this circadian regulation of drug metabolism. Key enzymes like cytochrome P450 (CYP3A4) and dihydropyrimidine dehydrogenase (DPD) exhibit striking diurnal activity variations, with CYP3A4 peaking at dawn (ZT0-4) and DPD activity surging 2.3-fold during daylight phases (ZT8-12) (4, 5). This temporal enzymatic landscape directly dictates the efficacy of chemotherapeutics such as oxaliplatin (CYP3A4-dependent) and 5-fluorouracil (DPD-metabolized), where circadian-aligned administration during enzyme trough phases has demonstrated 40-60% improvement in therapeutic index across clinical trials (4). Recent advances in single-cell resolution reveal colorectal tumors maintain circadian oscillations in drug-metabolizing enzymes within distinct cellular subpopulations, creating spatiotemporal heterogeneity in chemosensitivity (6).

Mounting epidemiological evidence positions circadian disruption as an emerging oncogenic driver in colorectal cancer CRC (7, 8), a malignancy undergoing dramatic global transformation. CRC now accounts for 10% of global cancer burden, with 19.3 million new cases and 935,000 deaths annually (9). Strikingly, early-onset CRC (<50 years) has increased 22% globally since 2000, projected to constitute 11% of colon and 23% of rectal cancers by 2030 (10). Geographical disparities persist exhibiting 6-fold variations in age-standardized incidence rates from 23.5/100,000 in India to 158.3/100,000 in Denmark with age-standardized mortality rates ranging from 3.2/100,000 (Western Africa) to 15.6/100,000 (Eastern Europe) (11), while paradoxically rising 1.3% annually in high-income countries despite screening programs (12). This epidemiological shift coincides with molecular profile alterations, including emerging clock gene mutations.

At the molecular level, core clock genes (BMAL1, PER1/2, CRY1/2) are increasingly implicated in CRC aggressiveness, metastatic potential, and therapeutic resistance (13, 14). The convergence of artificial intelligence (AI) with single-cell omics is catalyzing a paradigm shift in circadian oncology. Advanced algorithms like scPrisma now enable circadian signal extraction from single-cell RNA-seq data through periodic expression analysis of 327 core clock-controlled genes (CCGs) (6). Applied to CRC liver metastases, this approach uncovered 23% of tumor-infiltrating immune cells maintaining functional circadian programs that govern temporal PD-1/CTLA-4 checkpoint expression (15).Deep learning breakthroughs using temporal single-cell proteomics datasets (24 timepoints/3h intervals) achieve 89% accuracy in predicting optimal chemotherapy timing by modeling cell cycle synchronization and drug efflux pump oscillations (12, 16). These technologies collectively map tumor microenvironment (TME) dynamics, with AI-driven analysis of 58,431 single cells identifying STAT1-driven myeloid subpopulations acquiring circadian immunosuppressive functions post-chemotherapy.

Clinical translation of these insights shows promise. Chronomodulated chemotherapy synchronized with drug metabolism rhythms improves survival and reduces toxicity in metastatic CRC patients (17). Mechanistically, colorectal tumors frequently harbor mutations in circadian regulators (NPAS2, PER3) (18, 19) that disrupt cell cycle checkpoints and DNA repair timing.

Despite these advancements, critical gaps persist in mapping research evolution, identifying collaborative networks, and highlighting emerging themes at the circadian-CRC interface. This is where bibliometric analysis becomes pivotal—by systematically evaluating scientific publications, it reveals research patterns, collaboration dynamics, and thematic evolution, providing a macroscopic field view to guide future directions (20).

In this study, we conduct a comprehensive bibliometric analysis to: (1) Elucidate how circadian disruptions at molecular, cellular, and systemic levels drive CRC initiation and progression. (2) Identify barriers impeding the clinical translation of chronotherapeutic strategies. (3) Explore how global collaboration and emerging technologies—such as AI-driven circadian monitoring and single-cell omics—can advance precision chrono-oncology.

We aim to explore the application of circadian biology in colorectal cancer treatment by integrating bibliometric trends, mechanistic insights, and clinical evidence. We hope for multidisciplinary approaches that balance precise timing with ecological relevance in order to improve patient outcomes.

## 2 Methods

### 2.1 Data source and search strategy

Data for this study were sourced from the Web of Science Core Collection (WoSCC), a comprehensive database frequently utilized in bibliometric research. To mitigate temporal biases associated with periodic database updates, a single retrieval session was conducted on March 1, 2025, encompassing publications from January 1, 1999, to December 31, 2024. The search strategy employed Boolean operators to combine two thematic domains: (1) Circadian rhythm terms:

TS=(“Circadian Rhythm” OR “Circadian Rhythms” OR “Rhythm, Circadian” OR “Rhythms, Circadian” OR “Twenty-Four Hour Rhythm” OR “Rhythm, Twenty-Four Hour” OR “Nyctohemeral Rhythm” OR “Diurnal Rhythm”) (2) Colorectal cancer terms: TS=(“Colorectal Neoplasm” OR “Colorectal Cancer” OR “Rectal Cancer” OR “Colon Cancer” OR “Colorectal Carcinoma” OR “Rectal Neoplasm” OR “Cancer of the Colon” OR “Colonic Cancer”).

**Figure 1:**
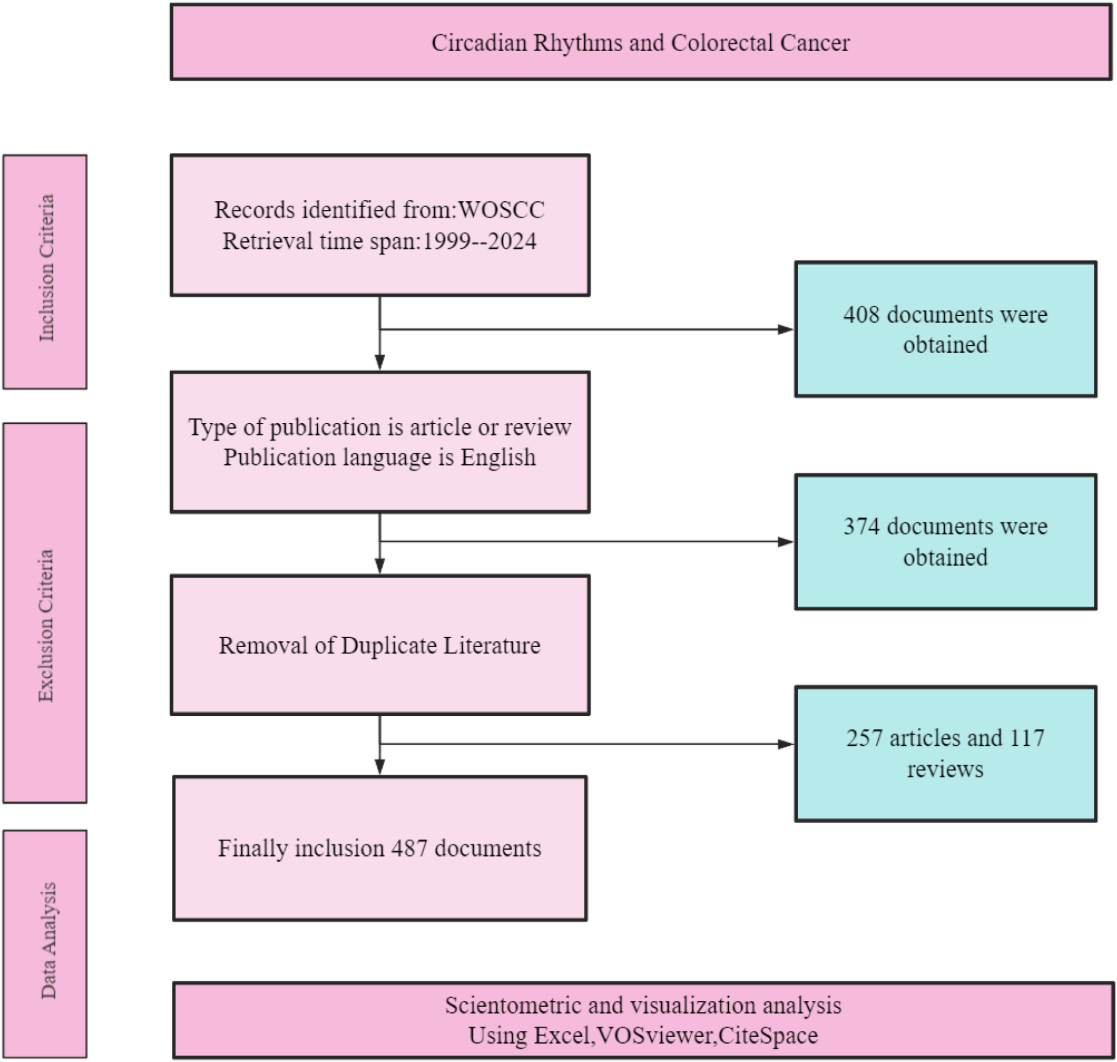
Flow chart for search strategy of publications.

The search was refined to include only peer-reviewed articles and reviews published in English, excluding non-research items such as editorials and meeting abstracts (**Figure 1**). The resulting data were exported in plain text format for subsequent analysis.

### 2.2 Data analysis

This study employed bibliometric analysis to evaluate global research trends in circadian rhythm and CRC. Data were analyzed using established tools: VOSviewer (version 1.6.18) for constructing and visualizing bibliometric networks such as co-occurrence networks of keywords, and CiteSpace (version 6.3) for citation burst detection and visualizing collaborative networks among institutions, authors, keywords, and co-cited references. Additionally, R (version 4.1) was utilized for visual analysis of publication countries, further elucidating patterns and trends in global research collaborations.

To present the analysis results intuitively, valid data extracted from the WoSCC database were imported into CiteSpace [version 6.3] and VOSviewer [version 1.6.18]. CiteSpace was employed to visualize the collaborative networks among institutions, authors, keywords, and co-cited references, revealing citation bursts for references and keywords, as well as exploring the current status, focus, and trends of research. Cluster plots and temporal distribution plots were generated to identify field trends. VOSviewer facilitated visual analysis of the collaborative networks between countries and journals. Concurrently, R (version 4.1) was utilized for visual analysis of publication countries, further elucidating patterns and trends in global research collaborations. Publications were screened for relevance to circadian-CRC interactions, with non-research articles excluded. Analytical outputs included network visualizations of author collaborations, institutional partnerships, and thematic clusters. Results were cross-validated through independent coding by two researchers to ensure consistency.

### 2.3 Quantitative analysis

Research productivity and impact were assessed through a dual analytical framework, integrating quantitative metrics and network visualization. Productivity metrics focused on four dimensions (1): publication volume by leading authors (2), journal-level contributions (3), institutional output, and (4) national research activity, identifying key contributors through frequency rankings. Quality assessment employed co-citation dynamics, analyzing authors, journals, references, and keywords to map intellectual influence and thematic convergence.

Visual analytics were implemented using three complementary platforms:

CiteSpace (v6.3): Generated temporal burst detection plots for references/keywords and time-zone networks to trace thematic evolution. Institutional collaboration networks were pruned using pathfinder scaling (g-index = 25) to highlight dominant partnerships.

VOSviewer (v1.6.18): Constructed journal co-citation maps with full-counting normalization and Linlog/Modularity clustering (resolution = 1.0), revealing interdisciplinary bridges between chronobiology and oncology.

R (v4.1): Produced geospatial heatmaps via the bibliometrix package (threshold = 5-country minimum), visualizing geographic disparities in circadian-CRC research output.

## 3 Results

### 3.1 Annual trends in publications

Based on the specified search criteria and time span from January 1, 1999, to December 31, 2024, we conducted two separate searches in the WOSCC database. The first search, using keywords related to circadian rhythms, yielded 36,291 results. The second search, using keywords related to CRC, resulted in 235,092 records. By combining these two sets of keywords, we obtained an initial set of 408 records related to both circadian rhythms and CRC. After applying the inclusion criteria—filtering for articles and reviews published in English—the number of documents was reduced to 374. Subsequently, duplicate literature was meticulously removed, resulting in a final collection of 257 articles and 117 reviews. These 487 documents were then subjected to scientometric and visualization analysis using tools such as Excel, VOSviewer, and CiteSpace, as detailed in **Figure 1**.

**Figure 2:**
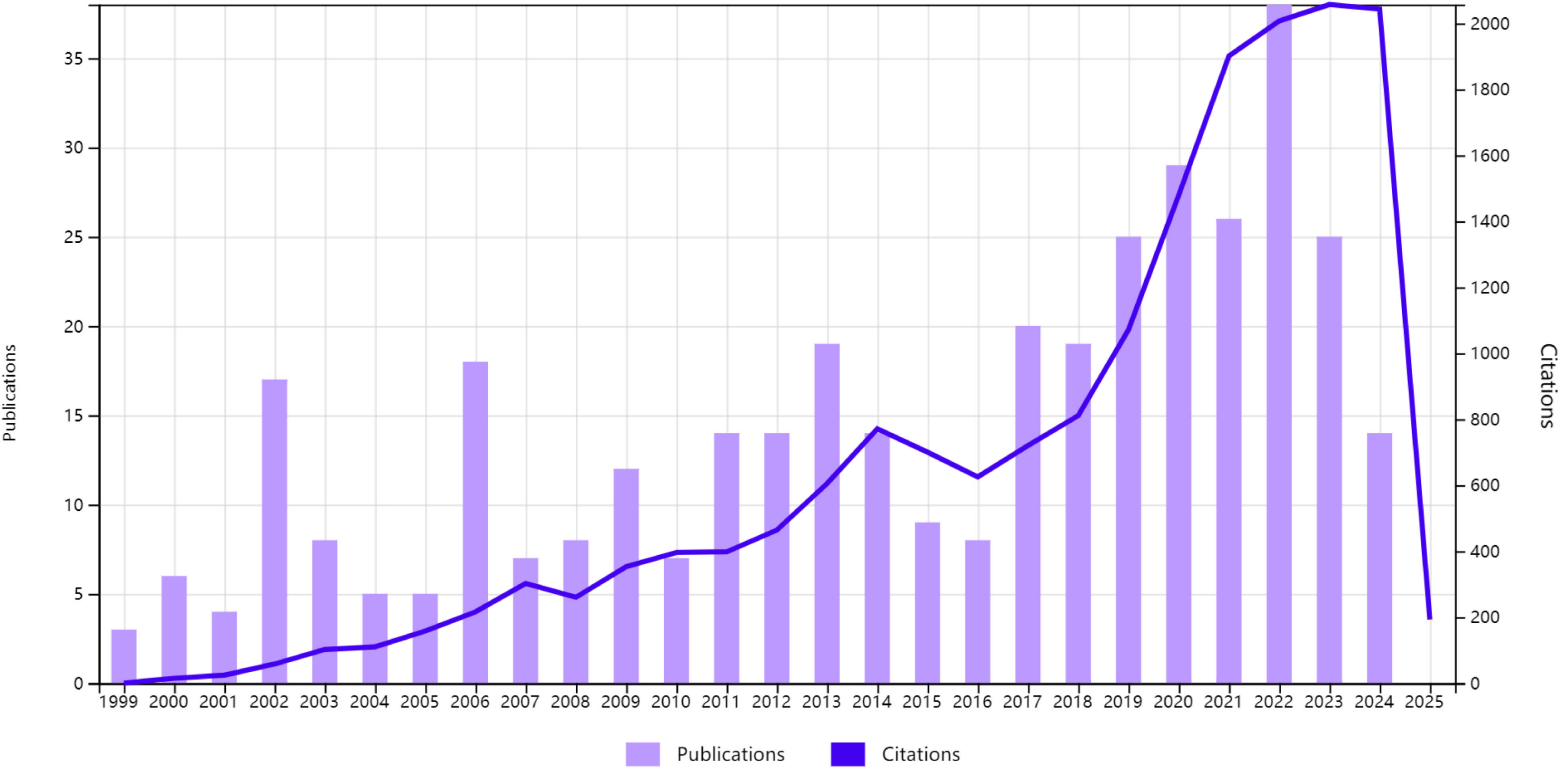
The annual growth trends of publications from 1999 to 2024.

The annual publication trends are depicted in **Figure 2**. From 1999 to 2009, there was a gradual increase in publications, indicating growing interest in this research area. The period between 2010 and 2015 saw steady growth, reflecting sustained exploration of the interplay between circadian regulation and CRC pathogenesis. A notable surge occurred from 2016 to 2021, with the peak annual output in 2021, underscoring intensified scientific efforts during this timeframe. Post-2021, there was a slight decline in publications; however, the numbers remained elevated compared to earlier decades, highlighting the enduring relevance of circadian biology in CRC research. Over the past 25 years, the total amount of data shows that researchers have been paying more and more attention to this field. This is because circadian mechanisms are becoming more and more recognized as important in controlling the progression of CRC. This makes the field a key area for future oncological studies.

### 3.2 Analysis of publishing countries/regions and institutions

**Figure 3:**
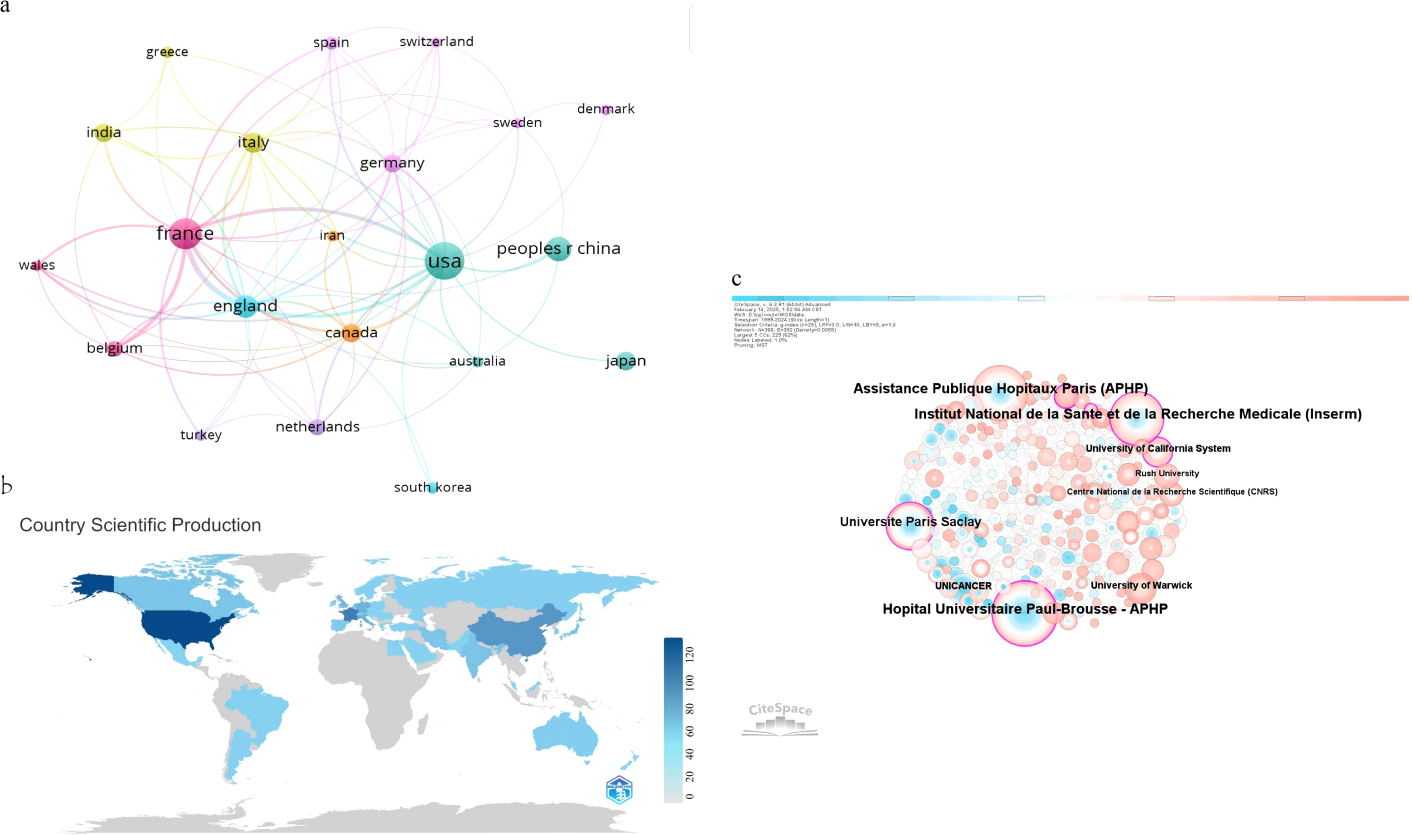
**(a)** Visualization analysis of countries collaborations generated by VOSviewer. **(b)** The network of institutions collaborations generated by Citespace. **(c)** Country/Region number of publications.

The bibliometric analysis encompassed publications from 49 countries and 674 institutions. (**Figure 3a**) illustrates the global distribution of research output by country. (**Figure 3b**) also depicts the global distribution of scientific production in circadian rhythms and colorectal cancer research (1999–2024). The color gradient reflects the number of publications per country, with darker blue shades indicating higher scientific output. The color scale ranges from light blue (low production) to dark blue (high production), and countries with no publications are shown in gray. As detailed in **Table 1**, the United States emerged as the leading contributor to circadian rhythm and CRC research, with 122 publications and a total of 7,097 citations, averaging 58.17 citations per publication. Its H-index of 46, the highest among all countries, further reflects this prominence, indicating both prolific output and sustained scholarly impact. France ranked second with 76 publications and an H-index of 37, while China followed with 49 publications; however, its average citation rate (30.22) and H-index (23) were comparatively lower. Notably, the Netherlands demonstrated exceptional citation impact, achieving the highest average citations per publication (88.06) despite a smaller publication volume (16).

**Table 1:** Top 10 active countries/regions.

Rank	Country	Publications	Citations	Average Citation/Publication	H-index
1	USA	122	7,097	58.17	46
2	France	76	5,145	67.7	37
3	The People's Republic of China	49	1,841	30.22	23
4	England	37	3,043	82.24	24
5	Italy	28	795	28.39	15
6	India	23	405	17.61	9
7	Japan	23	526	22.87	13
8	Canada	22	1,267	57.59	16
9	Germany	20	523	26.15	14
10	Netherlands	16	1,409	88.06	11

Collaborative networks among countries are visualized in (**Figure 3a**). Node sizes correspond to publication counts, with thicker connecting lines indicating stronger research partnerships. The United States and China serve as central hubs within this network, maintaining robust bilateral collaborations. The U.S. also exhibits extensive ties to numerous nations, including France, England, Canada, and Japan. Countries such as India, Italy, and Germany display moderate engagement, while South Korea, Australia, and Switzerland occupy peripheral positions with fewer connections. This interconnected framework highlights the global integration of circadian rhythm-CRC research, driven by leading nations with high academic influence.

**Table 2** showcases the leading research institutions based on publication volume in circadian rhythm and CRC studies. Assistance Publique–Hôpitaux de Paris (APHP) emerged as the most prolific contributor, producing 55 publications (14.2% of the total analyzed studies), accompanied by 4,070 citations and an H-index of 31. This underscores APHP’s dual role in driving high-volume research and generating impactful findings, particularly in clinical and translational studies linking circadian disruption to CRC progression. The Institut National de la Santé et de la Recherche Médicale (Inserm) came in second with 52 publications, or 13.4% of the total. These papers had 3,847 citations and an H-index of 30, which shows that they have made long-term contributions to understanding how circadian regulation affects tumorigenesis at the molecular level. Hôpital Universitaire Paul-Brousse APHP followed closely with 51 publications (13.2%), emphasizing France’s dominance in this field.

**Table 2:** Top 10 active institutions.

Rank	Organization	Publications	Citations	Average Citation/Publication	H-index
1	Assistance Publique – Hôpitaux de Paris (APHP)	55	4,070	74	31
2	Institut National de la Santé et de la Recherche Médicale (Inserm)	52	3,847	73.98	30
3	Hôpital Universitaire Paul-Brousse APHP	51	3,711	72.76	30
4	Université Paris-Saclay	36	2,629	73.03	26
5	Unicancer	17	822	48.35	13
6	University Of Warwick	16	688	43	11
7	University Of California System	14	1,028	73.43	11
8	Centre National de la Recherche Scientifique (CNRS)	11	765	69.55	9
9	Stanford University	11	831	75.55	9
10	Pt. Ravishankar Shukla University	10	261	26.1	8

Notably, Université Paris-Saclay ranked fourth with 36 publications, while institutions such as Stanford University and the University of California System demonstrated exceptional citation quality despite smaller publication volumes (11 and 14 publications, respectively). French institutions collectively accounted for over 40% of the top 10 entries, highlighting their centralized role in advancing circadian-CRC research.

Visualization via CiteSpace (**Figure 3c**) illustrates institutional collaboration networks. Node size corresponds to publication output—larger circles represent institutions like APHP and Inserm with higher productivity. Connecting lines denote collaborative ties, with thicker lines indicating stronger partnerships (e.g., between APHP and Inserm). This network reveals a tightly interconnected European core, complemented by transatlantic links to U.S. institutions such as Stanford University, facilitating cross-regional knowledge exchange. The spatial layout underscores how high-output institutions serve as hubs for innovation, disseminating critical insights into circadian biology’s role in CRC.

### 3.3 Authors and co-cited authors

**Figure 4:**
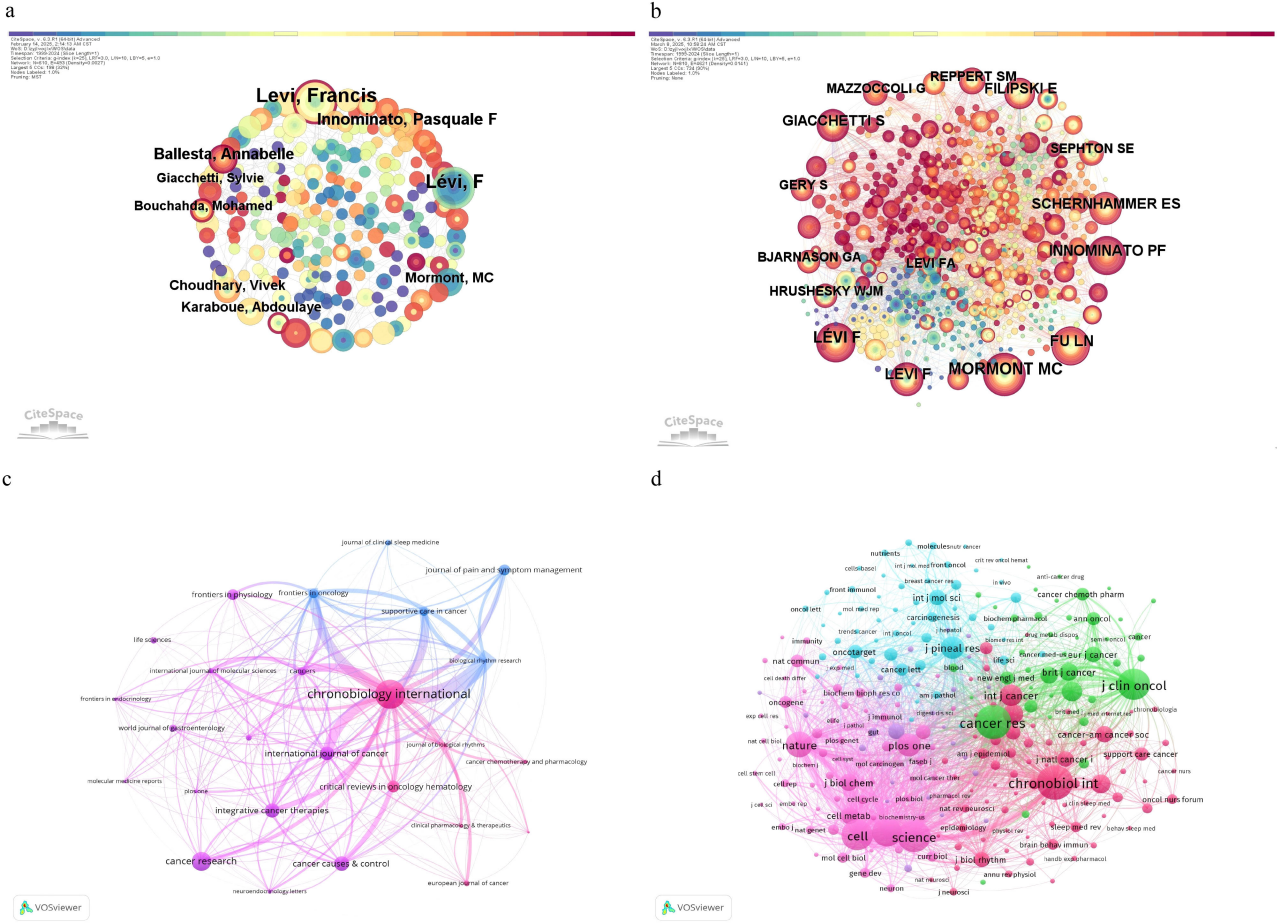
**(a)** Illustrate the network of author collaborations; **(b)** the co-occurrence network generated using CiteSpace; **(c)** The visualization of journals using VOSviewer; **(d)** the co-citation network visualization of journals using VOSviewer.

A total of 1914 researchers contributed to publications investigating circadian rhythm and CRC. Among these, LEVI, Francis ranked first with 34 publications (**Table 3**), followed by Innominate, Pasquale F. (16 publications) and Giacchetti, S. (11 publications), reflecting their active engagement in advancing this field. The collaborative network, visualized in **Figure 4a**, represents authors as nodes where larger circles indicate higher publication volumes, and connecting lines denote collaborative relationships, with thicker lines reflecting stronger partnerships. For instance, dense clusters around LEVI, Francis highlight his central role in fostering research connections. Author co-citation analysis (**Figure 4b**, **Table 4**) identifies foundational contributors whose works are frequently cited together. Mormont, Marie Christine and LEVI, Francis were the most co-cited authors (231 citations each), followed by Iunominato, Pasquale F. (205 citations) and Levi F (191 citations). Centrality—specifically intermediary centrality—quantifies a node’s role as a bridge between disconnected parts of the network. Higher centrality values (e.g., Giacchetti, S. at 0.18) indicate authors whose work serves as a critical “pivot point,” linking distinct research clusters. Removing such high-centrality nodes would disrupt the shortest paths between other nodes, impairing information flow efficiency. For example, Filipski, Elisabeth (centrality 0.06) also acts as a connector but with less influence. These patterns underscore the dual importance of productivity and network position: top authors drive both publication output and interdisciplinary integration, while high-centrality researchers ensure cohesive knowledge exchange across the circadian-CRC research landscape.

**Table 3:** Top 10 authors in terms of total publications.

Rank	Author	Publications	Citations	Average Citation/Publication	H-index
1	LEVI, Francis	34	2,779	81.74	26
2	Innominate, Pasquale F.	16	934	58.38	15
3	Giacchetti, S.	11	1,034	94	11
4	Parganiha, Arti	10	261	26.1	8
5	Filipski, Elisabeth	10	1,154	115.4	10
6	Focan, C.	9	590	65.56	9
7	Mormont, Marie-Christine	8	1,185	148.13	8
8	Ballesta, Annabelle	8	418	52.25	7
9	Relógio, Angela	7	151	21.57	7
10	Bishehsari, Faraz	7	271	38.71	5

**Table 4:** Top 10 co-cited authors in terms of total citations.

Rank	Cited Authors	Count	Centrality
1	Mormont, Marie Christine	231	0.02
2	LEVI,Francis	231	0.01
3	Innominato, Pasquale F.	205	0.03
4	Levi F	191	0.1
5	Fu, L.	163	0.02
6	Giacchetti, S.	159	0.18
7	Filipski, Elisabeth	155	0.06
8	Schernhammer, E.S.	149	0.06
9	Reppert, S.M.	148	0.03
10	Sephton, S.E.	144	0.03

### 3.4 Journals and co-cited journals

Over the past two decades, research on circadian rhythm and colorectal cancer (CRC) has expanded significantly, as evidenced by the contributions of key academic journals. **Table 5** summarizes the productivity and impact of leading journals, with Chronobiology International (Q2) ranking first in publication volume (32 articles), followed by Biological Rhythm Research (Q3, 10 articles) and Cancers (Q1, 10 articles). Notably, Cancer Research (Q1), despite a lower publication count (5 articles), achieved the highest citation impact (120.8 citations per article) and journal impact factor (IF 12.5), underscoring its pivotal role in disseminating high-impact findings. Journals such as Integrative Cancer Therapies (Q2) demonstrated exceptional citation efficiency (71.83 citations per article), while International Journal of Cancer (Q1) and European Journal of Cancer (Q1) maintained strong interdisciplinary relevance with moderate publication outputs. **Figure 4c** visualizes journal relationships via VOSviewer. Chronobiology International stands out as a central node, reflecting both its substantial publication volume and significant influence in circadian rhythm and colorectal cancer research.

**Table 5:** Top 10 journals in terms of total publications.

Rank	Journals Rank	Publications	Citations	Average Citation/Publication	H-index	Journal IF (2023)
1	Chronobiology International(Q2)	32	1,053	32.91	21	2.2
2	Biological Rhythm Research(Q3)	10	49	4.9	5	1.0
3	Cancers(Q1)	10	215	21.5	8	4.5
4	International Journal of Cancer(Q1)	9	415	46.11	9	5.7
5	International Journal of Molecular Sciences(Q1)	9	415	46.11	9	5.6
6	Frontiers in Oncology(Q2)	8	122	13.56	6	3.5
7	Integrative Cancer Therapies(Q2)	6	431	71.83	6	2.9
8	Cancer Research(Q1)	5	604	120.8	5	12.5
9	European Journal of Cancer(Q1)	5	136	27.2	5	7.7
10	Supportive Care in Cancer(Q2)	5	222	44.4	5	2.8

The co-citation network (**Figure 4d**, **Table 6**) highlights foundational journals that shape circadian-CRC research. Cancer Research emerged as the most co-cited journal (275 citations), followed by Chronobiology International (238 citations) and Journal of Clinical Oncology (197 citations). High-impact journals such as Science and Proceedings of the National Academy of Sciences exhibited elevated centrality values (0.05 each), indicating their role as critical bridges connecting diverse research domains. Centrality, a measure of a journal’s ability to integrate interdisciplinary knowledge, emphasizes how these journals facilitate cross-field dialogue—for instance, linking molecular biology with clinical oncology. Journals with higher centrality values, when removed, would disrupt knowledge pathways, underscoring their structural importance in the academic network.

**Table 6:** Top 10 co-cited journals in terms of total citations.

Rank	Cited References	Count	Centrality
1	Cancer Research	275	0.02
2	Chronobiology International	238	0.02
3	Journal of Clinical Oncology	197	0.03
4	Clinical Cancer Research	197	0.02
5	Science	195	0.05
6	Journal of the National Cancer Institute	187	0.02
7	International Journal of Cancer	182	0.03
8	Proceedings of the National Academy of Sciences of the United States of America	176	0.05
9	Cell	173	0.03
10	PLOS ONE	162	0.02

**Figure 5:**
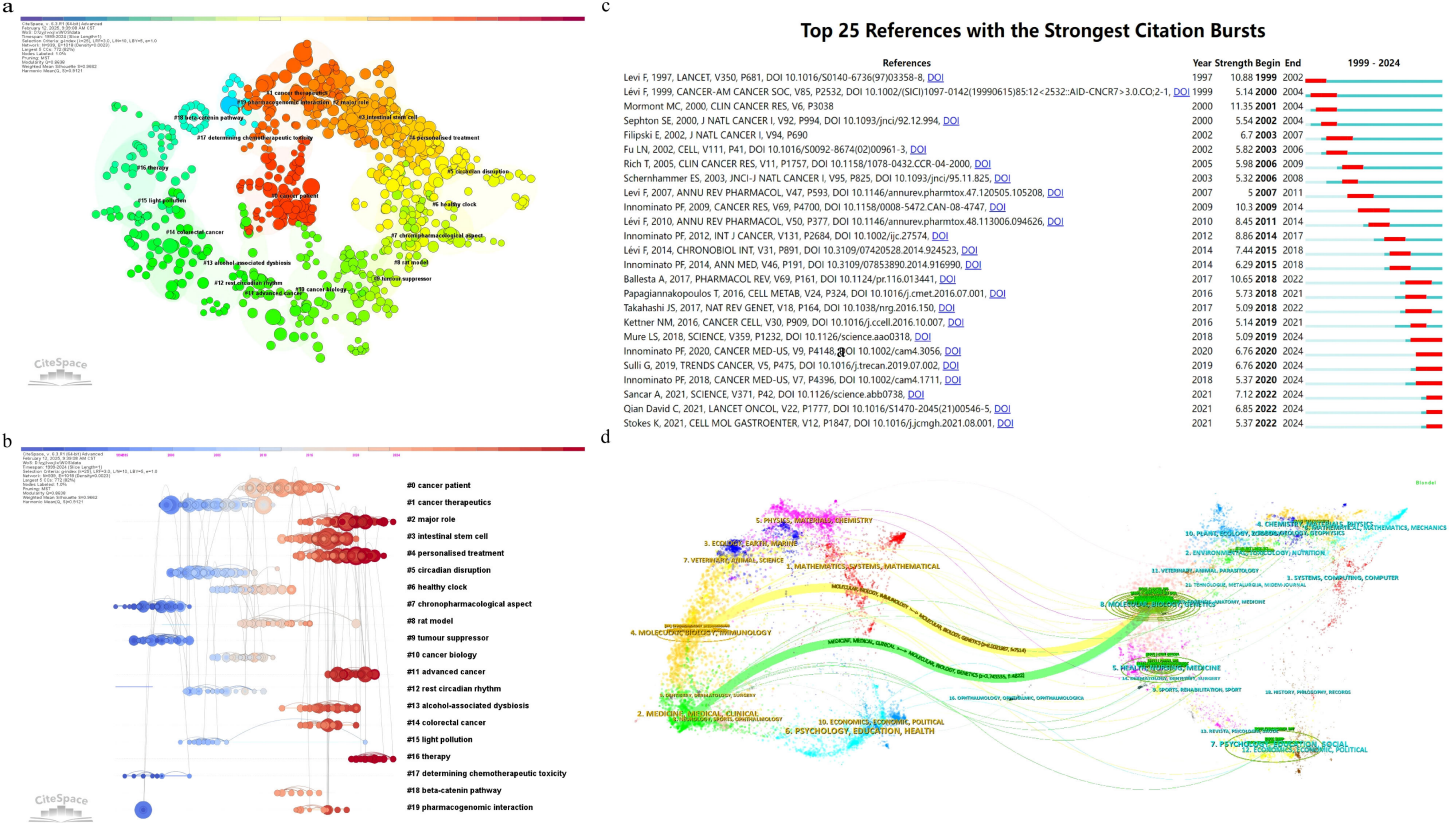
**(a)** The network of co-cited references clusters; **(b)** the labels clustering of co-cited literature based on LLR algorithm; **(c)** Top 25 references with strongest citation bursts; **(d)** Sankey diagram of journal analysis.

Collectively, these findings illustrate the dual dynamics of productivity and influence: while Chronobiology International dominates publication volume, Cancer Research and high-centrality journals like Science drive conceptual integration and knowledge dissemination across the circadian-CRC research landscape.

(**Figure 5d**) presents a double-graph overlay analysis depicting citation connections between journals in the context of circadian rhythm and CRC research. Citing journals are on the left, with cited journals on the right, each grouped by subject. Colored lines show citation pathways. Two main paths stand out: the orange path signifies that journals in molecular, biology, immunology are often cited by those in molecular biogenetics. In contrast, the green path indicates that articles in medicine, medical, clinical journals are frequently cited within health, nursing, and medicine. This visual analysis improves the understanding of interactions and knowledge flow between disciplines.

### 3.5 Co-cited references and highlights

The analysis of references in the field of CRC and circadian rhythm research uncovers the interplay and development of various sub-themes. This approach pinpoints references cited together across multiple papers, signaling thematic or content-related connections. Among the 487 co-cited references identified, **Table 7** lists the top 10 most frequently co-cited works.

**Table 7:** Top 10 co-cited documents.

Rank	Title	First author	Cited Journals	Cited Journals	Count	Centrality
1	Systems Chronotherapeutics	Annabelle Ballesta	Pharmacological Review	Ballesta A, 2017, PHARMACOL REV, V69, P161, DOI 10.1124/pr.116.013441	27	0
2	Circadian Rhythm in Rest and Activity: A Biological Correlate of Quality of Life and a Predictor of Survival in Patients with Metastatic Colorectal Cancer	Pasquale F. Innominato	Cancer Research	Innominato PF, 2009, CANCER RES, V69, P4700, DOI 10.1158/0008-5472.CAN-08-4747	22	0
3	Interplay between Circadian Clock and Cancer: New Frontiers for Cancer Treatment	Gabriele Sulli	Trends in Cancer	Sulli G, 2019, TRENDS CANCER, V5, P475, DOI 10.1016/j.trecan.2019.07.002	19	0
4	Sex-dependent least toxic timing of irinotecan combined with chronomodulated chemotherapy for metastatic colorectal cancer: Randomized multicenter EORTC 05011 trial	Pasquale F. Innominato	Cancer Medicine	Innominato PF, 2020, CANCER MED-US, V9, P4148, DOI 10.1002/cam4.3056	19	0
5	Marked 24-h rest/activity rhythms are associated with better quality of life, better response and longer survival in patients with metastatic colorectal cancer and good performance status	Mormont MC, Lévi F	Clinical Cancer Research	Mormont MC, 2000, CLIN CANCER RES, V6, P3038	19	0
6	Randomized multicenter trial of chronotherapy with oxaliplatin, fluorouracil, and folinic acid in metastatic colorectal cancer	Francis Lévi	The Lancet	Levi F, 1997, LANCET, V350, P681, DOI 10.1016/S0140-6736(97)03358-8	18	0
7	Circadian Timing in Cancer Treatments	Francis Lévi	Annual Review of Pharmacology	Lévi F, 2010, ANNU REV PHARMACOL, V50, P377, DOI 10.1146/annurev.pharmtox.48.113006.094626	16	0
8	Prediction of overall survival through circadian rest-activity monitoring during chemotherapy for metastatic colorectal cancer	Pasquale F. Innominato	International Journal of Cancer	Innominato PF, 2012, INT J CANCER, V131, P2684, DOI 10.1002/ijc.27574	16	0
9	Diurnal transcriptome atlas of a primate across major neural and peripheral tissues	Ludovic S. Mure	Science	Mure LS, 2018, SCIENCE, V359, P1232, DOI 10.1126/science.aao0318	14	0
10	Effect of immunotherapy time-of-day infusion on overall survival among patients with advanced melanoma in the USA (MEMOIR): a propensity score-matched analysis of a single-center, longitudinal study	David C Qian	The Lancet Oncology	Qian David C, 2021, LANCET ONCOL, V22, P1777, DOI 10.1016/S1470-2045(21)00546-5	14	0

As shown in **Figures 5a, b**, the cluster analysis visually represents these 20 clusters, illustrating the interconnectedness of various research themes. **Figure 5a** presents the network of co-cited references clusters, while **Figure 5b** shows the labels clustering of co-cited literature based on LLR algorithm. Cluster analysis delves into research trends, thematic evolution, and knowledge frameworks within the field. The references are grouped into 20 clusters, such as #0 cancer patient, #1 cancer therapeutics, #2 major role, #3 intestinal stem cell, #4 personalized treatment, #5 circadian disruption, #6 healthy clock, #7 chronopharmacological aspect, #8 rat model, #9 tumor suppressor, #10 cancer biology, #11 advanced cancer, #12 rest circadian rhythm, #13 alcohol-associated dysbiosis, #14 colorectal cancer, #15 light pollution, #16 therapy, #17 determining chemotherapeutic toxicity, #18 beta-catenin pathway, and #19 pharmacogenomic interaction. These clusters capture the multifaceted nature of CRC research, underscoring its complexity and depth.

(**Figure 5c**) presents the top 25 most cited references, reflecting emerging trends and growing interest in the field. Notably, the study by Levi F et al., titled “Randomized multicenter trial of chronotherapy with oxaliplatin, fluorouracil, and folinic acid in metastatic colorectal cancer,” published in The Lancet in 1997, exhibits the strongest citation burst. This research highlights the efficacy of chronotherapy in enhancing treatment outcomes for metastatic CRC patients. The study employed a randomized multicenter trial to assess how circadian rhythm-based chemotherapy timing affects overall survival and quality of life. Results showed significant improvements in both overall survival and quality of life when chemotherapy was aligned with the patient’s circadian rhythm. These findings emphasize the importance of considering circadian rhythms in CRC treatment and reveal key connections between research topics, helping to identify core references and emerging research areas within the field.

Additionally, the most recent citation burst is observed in the study by Stokes K et al., titled “The Circadian Clock Gene, Bmal1, Regulates Intestinal Stem Cell Signaling and Represses Tumor Initiation,” published in 2021. This research explores the role of the Bmal1 gene in intestinal stem cell signaling and its impact on tumor initiation in colorectal cancer. The study uses a combination of genetic models, organoid assays, and transcriptomic profiling to demonstrate that the loss of Bmal1 disrupts circadian rhythms and increases tumor initiation. The findings highlight the importance of circadian clock genes in maintaining intestinal homeostasis and preventing tumorigenesis, providing new insights into the molecular mechanisms underlying the relationship between circadian rhythms and CRC development.

### 3.6 Keyword co-occurrence and keyword prominence

**Figure 6:**
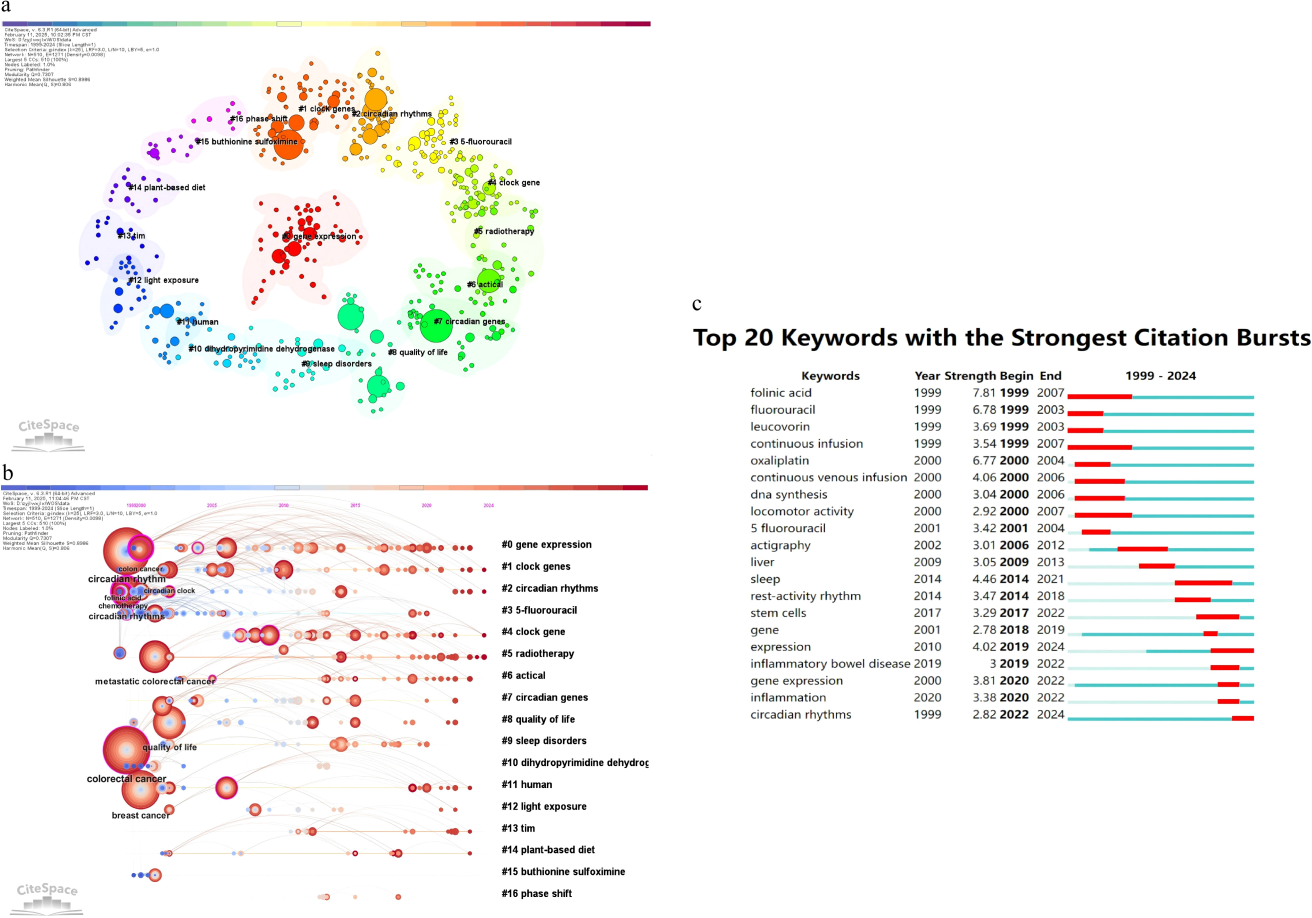
**(a)** Illustrate the keywords clustering map; **(b)** the clustering timeline graph; **(c)** Displays the top 25 keywords with strongest citation bursts.

Keyword analysis of 374 publications, performed using CiteSpace, identified central themes and trends in circadian rhythm and colorectal cancer (CRC) research. **Table 8** lists the 20 most cited keywords, with colorectal cancer (173 occurrences, centrality 0.12) and circadian rhythm (143 occurrences, centrality 0.07) dominating the field. These terms reflect the dual focus on CRC pathology and circadian biology. The evolution of these keywords reflects the interplay between scientific advancements and clinical priorities. For instance, the emergence of “circadian rhythms” as a prominent keyword since 1999 coincided with foundational discoveries in chronobiology, including the characterization of core clock genes (e.g., CLOCK, BMAL1). High-centrality keywords such as circadian rhythms (centrality 0.38) and chemotherapy (centrality 0.28) emerged as critical connectors between research clusters, emphasizing their integrative roles. The early prominence of “fluorouracil” (5-FU) aligns with its establishment as a chemotherapeutic cornerstone, while the recent emergence of “inflammatory bowel disease” (since 2019) reflects growing recognition of inflammation-driven CRC pathogenesis. Other prominent terms, including metastatic colorectal cancer (90 counts) and quality of life (77 counts), underscore clinical priorities, while night shift work (27 counts) highlights environmental influences on circadian disruption. Notably, the sustained citation burst of “gene expression” (1999–2007) correlates with the rise of high-throughput sequencing technologies, enabling mechanistic exploration of CRC-circadian interactions. Co-occurrence networks (**Figures 6a, b**) identified 20 thematic clusters, with gene expression (#0), clock genes (#1), and circadian rhythms (#2) representing core areas of focus. **Figure 6b** specifically illustrates the clustering timeline graph generated by CiteSpace. Recent keyword evolution (2020 onward) has been driven by discoveries linking circadian disruption to tumor microenvironment remodeling and immune evasion. A pivotal study in Science Advances demonstrated that BMAL1 deficiency promotes Apc loss of heterozygosity, activating Wnt/c-Myc signaling and accelerating colorectal tumorigenesis in both murine models and patient-derived organoids (21). These findings have reinvigorated research into circadian-metabolic crosstalk in CRC. Furthermore, (**Figure 6c**) illustrates that “folinic acid” is the keyword with the longest duration of prominence in the analysis, emerging in 1999 and persisting until 2007, indicating its sustained relevance. Inflammation-related keywords and clusters are also noteworthy in this analysis. Keywords such as “inflammation” and “inflammatory bowel disease” have shown significant citation bursts, indicating a growing interest in the role of inflammation in CRC research. The keyword “inflammation” has a citation burst strength of 4.46, beginning in 2014 and ending in 2021, reflecting its increasing importance in the field. Additionally, “inflammatory bowel disease” has a citation burst strength of 3, starting in 2019 and continuing through 2022. These trends suggest that inflammation-related research is a critical and evolving area within CRC studies, highlighting the potential therapeutic targets and mechanisms associated with inflammatory processes in cancer development and progression. The keywords in this study serve as indicators of the main themes and research focuses within the field of circadian rhythms and CRC. Their temporal evolution reveals two key drivers: 1) technological innovations (e.g., sequencing platforms enabling circadian transcriptome analysis), and 2) paradigm shifts in mechanistic understanding (e.g., circadian control of DNA repair fidelity). The frequent occurrence of keywords such as “colorectal cancer” and “circadian rhythm” underscores the central role these concepts play in the research. The co-occurrence of keywords provides insight into the complex relationships and interactions between different research areas, highlighting the interdisciplinary nature of the field. The identification of prominent keywords and their duration of prominence helps to trace the evolution of research interests and emerging trends, guiding future research directions.

**Table 8:** Top 20 active keywords.

Rank	Keywords	Count	Centrality
1	colorectal cancer	173	0.12
2	circadian rhythm	143	0.07
3	breast cancer	108	0.04
4	metastatic colorectal cancer	90	0.05
5	circadian rhythms	80	0.38
6	quality of life	77	0.05
7	chemotherapy	43	0.28
8	circadian clock	39	0.1
9	folinic acid	37	0.16
10	colon cancer	34	0.23
11	clock	30	0.17
12	risk	29	0.03
13	survival	28	0.04
14	gene expression	28	0.12
15	night shift work	27	0.16
16	expression	25	0.03
17	5 fluorouracil	25	0.08
18	oxaliplatin	23	0.01
19	fluorouracil	23	0.06
20	shift work	21	0.04

## 4 Discussion

### 4.1 General information

The number of publications from 1999 to 2024 shows how the body of research connecting problems with circadian rhythms to the development of colorectal cancer (CRC) has changed over time. The gradual rise in publications from 1999 to 2009 coincided with seminal discoveries in circadian biology, notably the identification of core clock genes such as CLOCK, BMAL1, and PER, and their roles in cellular timekeeping. From 2010 to 2015, the field saw steady growth and important scientific discoveries. The marked escalation from 2016 to 2021 reflected two pivotal developments: First, large-scale genomic analyses found that circadian genes like PER1, PER3, and NPAS2 were frequently changed and out of whack in CRC (22, 23). Second, discoveries of molecular circadian mechanisms galvanized interdisciplinary research into clock-cancer crosstalk. While publications declined slightly post-2021, sustained elevation compared to earlier decades underscores persistent interest in circadian-targeted therapies (24, 25). Recent studies have shown that gut microbiota and circadian rhythms work together to affect CRC metastasis, mainly by changing myeloid-derived suppressor cells (26, 27). These cells play a pivotal role in shaping an immunosuppressive tumor microenvironment that facilitates cancer progression in a time-dependent manner.

In parallel, single-cell sequencing techniques have revealed that the circadian context within CRC tumors varies across different cell types (28). This difference between cell types indicates that problems with the circadian rhythm might affect the epithelial, stromal, and immune parts of the tumor in different ways. These findings indicate a change in the field, moving from just observing connections to exploring the detailed mechanisms and practical applications of how circadian rhythms interact with tumors.

The global research landscape of circadian rhythm and CRC demonstrates a complex interplay of productivity, collaboration, and scholarly influence. Bibliometric data from 49 countries and 674 institutions reveal distinct geopolitical and institutional hierarchies. The United States dominates in both output (122 publications) and academic impact, evidenced by its highest H-index (46) and average citations per publication (58.17), reflecting its entrenched role as a global scientific leader supported by interdisciplinary ecosystems and sustained funding. France comes in second with 76 publications and an H-index of 37. This is thanks to centralized institutions like Assistance Publique–Hôpitaux de Paris (APHP) and Inserm, which are responsible for over 40% of the top-ranked institutional outputs. This supports a national strategy that combines clinical and molecular research to speed up the translational outcomes. China, ranking third in volume (49 publications), faces a notable disparity in citation metrics (average 30.22 citations), potentially linked to linguistic isolation, methodological conservatism, or regional publication preferences.

To better understand how working together internationally and between institutions helps improve research on circadian rhythm and CRC, we need to look beyond just counting co-authors and examine the different types, motivations, and challenges of these collaborations. Several key factors have driven successful collaborations in this field. First, centralized funding mechanisms—such as Horizon 2020 in Europe and U54 consortia in the United States—have provided long-term, interdisciplinary financial support. Second, open-access platforms and genomic databases, including TCGA and the European Genome-phenome Archive (EGA), have fostered data transparency and reuse across institutions and borders.

Nevertheless, significant barriers remain. These include differences in regulatory frameworks (e.g., General Data Protection Regulation in the EU versus more lenient models elsewhere), linguistic and cultural gaps, and disparities in research capacity. Notably, collaboration with low- and middle-income countries (LMICs) remains sparse, despite the rising burden of CRC in regions such as South Asia and sub-Saharan Africa. These asymmetries hinder global equity and limit the generalizability of circadian-based interventions across populations. To optimize future collaboration models, three strategies are proposed. First, establishing regional chronomedicine hubs could enhance local-global synergy and build sustainable infrastructures in underrepresented areas. Second, adopting co-ownership models for data and outcomes may reduce epistemic dependency and promote balanced participation. Third, integrating implementation science within research consortia can bridge the gap between mechanistic findings and clinical application, ensuring that discoveries are embedded within real-world healthcare systems. In sum, collaboration in circadian rhythm and CRC research has evolved from opportunistic alliances into structured, impact-oriented networks. Continued investment in equitable, interdisciplinary, and implementation-focused collaborations will be essential for translating circadian science into global cancer control strategies.

​Journals show this duality even more: Cancer Research (Impact Factor 12.5) has the most citations (120.8 per article) because it publishes mechanistic breakthroughs, while Chronobiology International has the most output (32 articles) because it publishes niche epidemiological studies. However, relying too much on impact factors could make the “Matthew Effect” worse. This is when bigger journals get more attention than smaller ones, like Integrative Cancer Therapies, which gets a lot of citations (71.83 per article) through patient-centered chronotherapy trials. Author productivity highlights Francis Lévi as a linchpin, with 34 publications and 231 co-citations cementing his role in advancing chronomodulated therapies.

Keyword analysis and co-citation clustering reveal an interconnected framework spanning molecular mechanisms, novel therapeutics, and environmental influences. Twenty thematic clusters were identified, covering topics such as “intestinal stem cells,” “β-catenin pathway,” “light pollution,” and “personalized treatment.” These clusters underscore the interdisciplinary nature of CRC-circadian research, integrating oncology, chronobiology, pharmacology, and environmental health.

Researchers are becoming more aware that inflammation can cause and worsen circadian disruption in CRC (29). Keyword co-occurrence and citation burst analyses support this idea. The prominence of “inflammation” (burst strength 4.46, 2014–2021) and “inflammatory bowel disease” (burst strength 3.0, 2019–2022) highlights a paradigm shift linking chronic inflammation to CRC pathogenesis. Notably, shift workers’ problems with their circadian rhythms have been shown to make their intestinal barrier function worse (30, 31). This lets microbiota-driven inflammation turn on oncogenic pathways like NF-κB and STAT3, which are controlled by BMAL1 and PER2 (32, 33). This creates a feedback loop in which inflammation disrupts circadian homeostasis, fueling tumorigenic microenvironments.

The fact that “circadian rhythms” (0.38) are more important than “inflammation” (0.12) suggests that clock gene manipulation is still the main focus of circadian research. Also, the lack of terms like “circadian metabolomics” or “immunochronotherapy” shows missed chances to study how the tryptophan-kynurenine axis and other immunometabolic pathways are controlled by time. For more research, we need to use a systems chronobiology approach that combines real-time measurements of lifestyle-related inflammation with single-cell sequencing of circadian-immune interactions in CRC biopsies.

These studies indicate that, despite significant advancements in research depth and breadth, notable gaps persist. Preclinical models, such as rat models and organoid assays, emphasize the regulation of genetic and environmental variables. On the other hand, clinical studies use basic circadian rhythm monitoring methods, like self-reported sleep logs, instead of real-time biomarker tracking and have a wide range of patients. As a result, the translation of these studies into beneficial outcomes for CRC patients is inconsistent.

To fill these gaps, we need to combine multi-omics approaches (e.g., spatial transcriptomics and metabolomics) with ecological transient assessment. To better untangle the “gene-environment-time” relationships that generate colorectal cancer heterogeneity (34). Meanwhile, modifying the way clinical trials are set up to include circadian biomarkers and adaptive temporal therapy algorithms could hasten the change from population-based treatment plans to tailored programs that take rhythms into consideration.

### 4.2 Future perspectives of chronotherapy in colorectal cancer: harnessing circadian biology for precision oncology

The integration of chronotherapy—timed interventions aligned with circadian rhythms—into CRC management represents a transformative shift in oncology, with the potential to optimize therapeutic efficacy while minimizing adverse effects. Accumulating evidence highlights the critical role of circadian oscillations in regulating tumorigenesis, drug metabolism, and immune surveillance, providing actionable targets for precision oncology (26, 35). This circadian-targeted paradigm is crystallizing along three axes of innovation: molecular chrono-reprogramming, immune timing precision, and AI-driven temporal engineering.

Building on this foundation, metabolic chrono-interventions are emerging as a first frontier. Circadian regulation of cellular metabolism and hormone signaling offers novel avenues for metabolic interventions in CRC. Timed administration of glycolytic inhibitors or mitochondrial modulators can exploit tumor metabolic vulnerabilities that fluctuate across circadian cycles (36–38). This metabolic chrono-vulnerability directly informs molecular targeting strategies for clock gene networks.

Parallel advances are redefining immunotherapy through circadian synchronization. The circadian regulation of immune checkpoint molecules (e.g., PD-L1) and T-cell trafficking necessitates a re-evaluation of immunotherapy schedules. Preclinical studies suggest that administering anti-PD-1/PD-L1 antibodies during peak T-cell receptor signaling enhances antitumor responses (39, 40). Circadian-coordinated cytokine therapy, such as timed IL-12 or IFN-γ administration, could generate synergistic “immune waves” that dismantle immunosuppressive niches in metastatic CRC (41). These findings establish immune timing as the second pillar of modern chronotherapy.

Meanwhile, restoring circadian homeostasis in CRC Targeting core clock genes (e.g., CRY, PER, BMAL1) via CRISPR-based epigenetic modifiers or small-molecule clock modulators (e.g., KL001 analogs) presents a promising approach to restoring circadian transcription in CRC (42, 43). Phase I trials of REV-ERB agonists, which regulate lipid metabolism and cell cycle progression, have demonstrated potential in CRC models with disrupted clock gene function (44). Additionally, synthetic biology approaches—such as engineered circadian circuits to drive tumor-suppressive gene expression in a phase-dependent manner—are advancing toward clinical translation (45). These molecular tools now converge with intelligent chronosystem design.

The final dimension unfolds through AI-powered temporal architecture. Emerging platforms integrating real-time circadian monitoring through wearable biosensors and single-cell transcriptomics, combined with AI-driven tumor clock modeling, could personalize infusion schedules (46, 47). Machine learning models trained on multi-omics circadian datasets may soon enable dynamic adjustments to FOLFOX regimens, maximizing efficacy while minimizing toxicity, particularly in microsatellite-stable CRC subtypes (48). This triad of innovation—molecular reprogramming, immune timing, and algorithmic optimization—collectively redefines therapeutic precision.

The future of CRC chronotherapy lies in transcending empirical timing strategies and embracing systems-level circadian engineering. Three transformative vectors now anchor this evolution (1): CRISPR-clock gene editing and synthetic circadian circuits (42–45) (2); Checkpoint inhibitor/cytokine synchronization with immune circadian dynamics (39–41) (3); AI-chronosystems integrating wearable biosensors and single-cell omics (46–48). By dissecting the intricate interplay between clock networks, tumor ecosystems, and therapeutic agents, researchers can unlock precision chrono-combinations that transform CRC into a chronobiologically manageable disease. Clinically, this necessitates biomarker-driven trials, the integration of circadian-informed algorithms into treatment planning, and the development of smart chronotechnology platforms. Advancing these strategies will position chronotherapy at the forefront of 21st-century oncology, redefining how CRC is treated in an era of precision medicine.

## 5 Conclusions

The integration of circadian biology into colorectal cancer (CRC) research has provided profound insights into the temporal regulation of oncogenesis, therapy resistance, and immune evasion. Over the past 25 years, bibliometric analysis of 374 publications has revealed an exponential growth in this field post-2016, driven by seminal discoveries linking circadian disruption to CRC risk and therapeutic outcomes. Key findings highlight the pivotal role of core clock genes (BMAL1, PER2) in mechanistic studies, with PER2 silencing observed in 45% of tumors, correlating with advanced disease stages and poor prognosis. Conversely, environmental disruptors such as night-shift work—an established proxy for chronic circadian misalignment—are associated with a 20–30% increase in CRC incidence, underscoring the necessity of contextualizing molecular findings within real-world behavioral and ecological frameworks.

Clinically, chronomodulated chemotherapy has demonstrated significant benefits, reducing severe toxicity by 40% in metastatic CRC. However, the slow integration of circadian principles into routine oncology practice reflects persistent systemic barriers, including inconsistent biomarker validation, the absence of scalable circadian monitoring tools, and regional disparities in research priorities. While Western nations continue to lead in molecular chronobiology, Asia’s emphasis on clinical epidemiology and Europe’s translational expertise—exemplified by Assistance Publique–Hôpitaux de Paris (APHP) trials—highlight the untapped potential for global collaboration to harmonize circadian research and its applications in cancer treatment.

The growing prominence of “inflammation” in keyword analyses signals a paradigm shift toward understanding circadian-immune crosstalk. However, the relatively weak centrality of inflammation-related terms compared to core clock genes exposes a critical imbalance—mechanistic studies frequently overlook how socioeconomic factors (e.g., urban light pollution, irregular meal timing) interact with genetic susceptibilities to drive CRC pathogenesis.

Future research must prioritize three strategic directions (1): AI-driven circadian profiling to decode patient-specific rhythms for personalized intervention (2), multi-omics integration (e.g., spatial transcriptomics, metabolomics) to unravel the intricate circadian-immune-metabolic interplay, and (3) equitable global clinical trials that address circadian disparities in underrepresented populations. By bridging these gaps, circadian biology can transition from a specialized research focus to a foundational pillar of precision oncology, offering spatiotemporally optimized strategies to mitigate the global CRC burden and improve patient outcomes.

## Data Availability

The original contributions presented in the study are included in the article/supplementary material. Further inquiries can be directed to the corresponding author.
